# Exploring the educational needs of patients with systemic vasculitis using the educational needs assessment tool

**DOI:** 10.1093/rap/rkac062

**Published:** 2022-08-11

**Authors:** Sara Brolin, Elisabet Welin, Björn Lövström, Annette Bruchfeld, Iva Gunnarsson, Susanne Pettersson

**Affiliations:** Department of Rheumatology, Karolinska University Hospital; Department of Medicine Solna, Karolinska Institutet, Stockholm; School of Health Sciences, Örebro University, Örebro; Department of Rheumatology, Karolinska University Hospital; Department of Medicine Solna, Karolinska Institutet, Stockholm; Department of Health, Medicine and Caring Sciences, Linköping University, Linköping; Department of Renal Medicine, Karolinska University Hospital; Department of Clinical Science, Intervention and Technology, Karolinska Institutet, Stockholm, Sweden; Department of Rheumatology, Karolinska University Hospital; Department of Medicine Solna, Karolinska Institutet, Stockholm; Department of Rheumatology, Karolinska University Hospital; Department of Medicine Solna, Karolinska Institutet, Stockholm

**Keywords:** ANCA-associated vasculitis, educational needs, nursing, health literacy, outcome measures, patient experience

## Abstract

**Objectives:**

Knowledge and health literacy enable patients to monitor symptoms and disease impact. Educational needs have previously been explored in rheumatology, but scarcely for patients with ANCA-associated vasculitis (AAV). The aim of the study was to assess the educational needs among patients with AAV using the educational needs assessment tool (ENAT).

**Methods:**

This was a cross-sectional observational study including adults with AAV. Educational needs were captured by ENAT. Total ENAT (0–117 points, with higher numbers indicating higher educational need) and the seven domains (managing pain, movement, feelings, disease process, treatment, self-management and support systems) were explored regarding sex, age, education, diagnosis, disease duration and disease activity. To compare domains, a percentage response (0–100%) was calculated.

**Results:**

One hundred and seventy-eight individuals (50% men; 34% with disease duration ≤2 years) were included. The total ENAT mean was 66.5 (s.d. 26.6; 57%), with domains as follows: disease process, 78%; self-management, 69%; treatments, 64%; feelings, 56%; managing pain, 48%; support systems, 47%; and movement, 41%. Higher educational needs were found among women in the domains movement, feelings and disease process and in total ENAT (all *P* < 0.04) compared with men. Higher educational needs were also seen in patients with disease duration ≤2 years regarding disease process, self-management and support systems and in total ENAT compared with patients with longer disease duration (all *P* < 0.03).

**Conclusion:**

This study revealed great educational needs among AAV patients. Some groups expressed higher needs (women and those with shorter disease duration). Increased education for patients with AAV might lead to improved self-care and treatment adherence.

Key messages

- Patients with ANCA-associated vasculitis express a distinctive need for education.
- The greatest demand for education is concerning the disease process, self-management and treatments.
- Women and those with shorter disease duration express a greater need for education than others.

## Introduction

ANCA-associated vasculitis (AAV) is a rare but potentially life-threatening disease, with a high morbidity if left untreated [1]. It consists of three disease entities [granulomatosis with polyangiitis (GPA), microscopic polyangiitis (MPA) and eosinophilic GPA (EGPA)], which share clinical manifestations, treatment and outcome. However, the clinical manifestations can vary substantially, both between individuals and during the disease course in single patients. Adequate education regarding the disease is therefore of importance and can help patients to detect early flares, cope with symptoms and stay adherent to treatment.

Low health literacy is associated with worse medical outcomes [2]. EULAR, the British Society for Rheumatology (BSR) and British Health Professionals in Rheumatology (BHPR) recommend that patients receive education on their disease to increase their ability to self-monitor potential symptoms [3], recognize early signs and symptoms of relapse to increase treatment compliance, achieve a long-term positive outcome and prevent increased morbidity [1]. Furthermore, AAV patients who are educated regarding medications and potential side effects report better health-related quality of life [4].

The development of internet sources and on- and off-line patient support groups can provide information, advice and peer support to share and increase knowledge, but they can, potentially, also confer a risk for inaccurate information [1]. Additionally, there are indications that patients with AAV prefer to receive information from health-care professionals rather than the internet or patient groups [5]. Nevertheless, patients can find the information provided by health-care professionals difficult to comprehend [6], and studies have shown that patients with AAV require information tailored to different stages of the disease [7]. Of note, AAV patients who reported having received insufficient information about medication were shown to have lower adherence to medication [8]. Patients who perceive information or self-care advice to be of importance have been shown to have a higher adherence to treatment [6].

The educational needs assessment tool (ENAT) survey was developed in England in the early 2000s to evaluate the educational needs of patients with RA [9]. The survey has been validated for seven different rheumatic diseases (RA, AS, FM, PsA, SLE, scleroderma and OA), and it has been cross-culturally adapted, translated and validated into several languages, including Swedish, and is considered generic [10–12]. Using ENAT in patient education has been shown to be of value to identify and prioritize the individual educational needs in persons with RA [13]. Studies on educational needs among AAV patients are, however, scarce.

The aim of the study was to use ENAT to investigate the educational needs of AAV patients and to explore differences related to sex, age, education, diagnosis, disease duration and disease activity.

## Methods

This is an observational study based on cross-sectional data collected between 2008 and 2019. Patients with AAV and a minimum age of 18 years were included consecutively from a rheumatology clinic at a the Karolinska University Hospital in Stockholm, Sweden. Patients with previous or new diagnosis were asked to participate. The AAV diagnostic groups were divided into subgroups (GPA, MPA,and EGPA) according to the European Medicines Agency (EMEA) algorithm, and all were ANCA positive at the time of diagnosis [14]. The data collection and the cohort have been described in a previous publication [15]. For the present study, additional exclusion criteria were added: insufficient knowledge of and inability to read and understand written Swedish.

The study complied with the Declaration of Helsinki and was approved by the regional ethical review board in Stockholm (Etikprövningsmyndigheten). Voluntary participation was emphasized in the study, and confidentiality was guaranteed. Each participant signed an informed consent form.

### Questionnaires regarding educational needs

At inclusion in the cohort, all participants were asked to answer the Swedish version of the ENAT questionnaire [10, 11]. The ENAT captures the patient’s educational needs in relationship to rheumatic diseases [9]. The first question, ‘Do you currently require information about something that can help you with your rheumatic disease?’, is answered yes/no, with an open follow-up question, ‘If yes, what?’, answered in free text.

The ENAT consists of seven domains (managing pain, movement, feelings, disease process, treatment, self-management and support systems); each contains four to seven questions (four-point Likert scale: not at all important = 0, a little important = 1, very important = 2 and extremely important = 3). The total ENAT score ranges between 0 and 117, with a higher score indicating greater need for education. The domain scores range from 0 to a maximum between 12 and 21. To facilitate comparisons between the domains, the percentage response (0–100%) was calculated for each domain. The total sum of the responses in the domain was divided by the maximum score for that domain [9]. Thus, 0% indicates that no education is requested, whereas 100% indicates the greatest need for education. The responses are presented as the mean percentage of the domain score.

Deductive content analysis was chosen to analyse the free text answers of the open question [16]. The ENAT was used as a matrix, and the written answers from the participants were sorted according to the ENAT domains. Extended answers were, if appropriate, categorized into several statements. Free text answers that could not be sorted according to the ENAT matrix were marked as ‘other’.

### Disease activity

The BVAS was used to assess disease activity [17] at the time point for inclusion in the study. Having zero points indicated no ongoing disease activity, whereas at least one point indicated signs of active disease.

### Subgrouping of variables

The participants were divided into four age groups: 18–30, 31–50, 51–70 and 71–85 years.

Primary school (0–12 years) and higher education (>12 years) were the two education groups. Given that primary school (9 years) is mandatory in Sweden and high school education (3 years) is customary, all education after high school can be considered higher education [18]. Disease duration was categorized into two groups: disease duration of ≤2 years and ≥3 years, based on the time of confirmed diagnosis of AAV.

### Statistics

Quantitative analyses were performed using the Statistical Package for the Social Sciences (SPSS v.24, SPSS, Chicago, IL, USA). Descriptive data are displayed as the mean with s.d. Student’s unpaired *t*-test was used to compare the ENAT scores for variables categorized into two groups (sex, education, disease duration and disease activity). One-way analysis of variance (ANOVA) was used to compare the ENAT scores for variables categorized into several groups (age groups and diagnosis). Values of *P* < 0.05 were considered statistically significant. When a single response or several responses to questions in a domain were missing, all the responses of that individual for that domain were excluded [19]. Internal consistency and reliability was determined by calculating Cronbach’s α and considered good if Cronbach’s α was >0.70 [20].

## Results

The study included 178 individuals, with an equal representation of men and women (Table 1). The median age was 61 (IQR = 20) years, and 33.7% had been diagnosed with AAV within 2 years before inclusion. The mean (s.d.) disease duration was 10.8 (15.3) years, with a range of 0–66 years. The median disease activity assessed with BVAS (0–63) was 2 (IQR = 9), ranging between 0 and 30, with 105 individuals (59%) having active disease as defined by a BVAS ≥ 0. Higher education was reported in 53% of the patients.

**Table 1:** Patient demographics and grouping variables, *n* = 178

Characteristic	Value
Age, mean (s.d.) (range), median (IQR), years	57.5 (15.7) (18–85), 61 (20)
18–43, *n* (%)	36 (20)
44–57, *n* (%)	38 (21)
58–64, *n* (%)	34 (19)
65–70, *n* (%)	38 (21)
71–85, *n* (%)	32 (18)
Sex, *n* (%)	
Male	89 (50)
Female	89 (50)
Diagnosis, *n* (%)	
GPA	141 (79)
MPA	24 (14)
EGPA	13 (7)
Disease duration, mean (s.d.) (range), median (IQR), years	10.8 (15.3) (0–66), 5 (10)
≤2 years, *n* (%)	60 (34)
≥3 years, *n* (%)	118 (66)
Disease activity (BVAS), mean (s.d.) (range), median (IQR)	5.7 (7.7) (0–30), 2 (9)
Active disease, *n* (%)	105 (59)
No disease activity, *n* (%)	73 (41)
Education^a^, mean (s.d.) (range), median (IQR), years	13.2 (3.9) (2–35), 13 (4)
Primary school and high school, *n* (%)	74 (42)
Higher education, *n* (%)	94 (53)

a Missing data, *n* = 10.

EGPA: eosinophilic granulomatosis with polyangiitis; GPA: granulomatosis with polyangiitis; IQR, interquartile range; MPA: microscopic polyangiitis.

### Self-reported educational needs

Sixty-seven of the 178 participants (38%) answered ‘yes’ to the single question, ‘Do you currently require information about something that can help you with your rheumatic disease?’. In this group, women (61%, *P* = 0.02) and those with a disease duration of ≥3 years (56%, *P* = 0.02) were overrepresented.

Sixty of those sixty-seven individuals (90%) also responded to the open-ended question. The written answers resulted in a total of 73 statements. All statements, except five, could be categorized into any of the seven ENAT domains (Table 2). The statements categorized as ‘other’ represented three patients who wrote that they were unsure if they had AAV, and two who wrote ‘everything’. Thus, the disease process (*n* = 31), self-management (*n* = 18) and treatments (*n* = 12) domains contained the most statements (Table 2).

**Table 2:** Internal consistency and categorization of free text response of educational needs^a^ in relationship to the educational needs assessment tool domains and relevance of the domains

ENAT domain	**Questions** ^b^	Cronbach’s α (*n* = 178)	Statements (*n* = 60)
Managing pain	6	0.90	1
Movement	5	0.91	3
Feelings	4	0.93	2
Disease	7	0.86	31
Treatments	7	0.91	12
Self-management	6	0.92	18
Support	4	0.88	1
Other	–	–	5
Total	39	0.97	73

a ‘Do you currently require information about something that can help you with your rheumatic disease?’ is answered yes/no, with the follow-up question, ‘If yes, what?’.

b Number of questions/items per ENAT domain.

ENAT: educational needs assessment tool.

### Educational needs captured by total ENAT

The total ENAT, which included all 39 items, resulted in Cronbach’s α = 0.97, and the domains ranged from 0.86 to 0.93 (Table 2).

Table 3 displays the results corresponding to the seven ENAT domains. The total ENAT (s.d.) was 66.5 (26.6) (57%).

**Table 3:** Comparisons of educational needs assessment tool domains, mean score (s.d.) and (mean percentage of the domain score)

Domain	All	Women (*n* = 89)	Men (*n* = 89)	Mean difference	*P*-value	Disease duration ≤2 years (*n* = 60)	Disease duration ≥3 years (*n* = 118)	Mean difference	*P*-value	Primary school and high school (*n* = 74)	Higher education (*n* = 94)	Mean difference	*P*-value
Managing pain, 0–18 (*n* = 174)	8.6 (5.2) (48%)	9.0 (5.3) (50%)	8.1 (5.0) (45%)	−0.9	0.234	9.1 (5.4) (50%)	8.3 (5.0) (46%)	−0.7	0.372	8.8 (5.0) (50%)	8.2 (5.1) (46%)	−0.6	0.433
Movement, 0–15 (*n* = 166)	6.2 (4.6) (41%)	7.5 (4.7) (50%)	4.9 (4.2) (33%)	−2.6	**<0.001**	6.6 (4.8) (44%)	6.0 (4.6) (40%)	−0.6	0.448	6.7 (4.6) (49%)	5.5 (3.2) (35%)	−1.2	**0.006**
Feelings, 0–12 (*n* = 170)	6.7 (3.9) (56%)	7.9 (3.8) (66%)	5.5 (3.7) (46%)	−2.4	**<0.001**	7.1 (4.2) (59%)	6.5 (3.8) (54%)	−0.6	0.326	7.0 (3.7) (60%)	6.3 (4.2) (53%)	−0.7	0.195
Disease process, 0–21 (*n* = 174)	16.4 (4.5) (78%)	17.1 (4.1) (81%)	15.7 (4.7) (75%)	−1.4	**0.041**	18.0 (3.2) (86%)	15.6 (4.8) (74%)	−2.4	**0.001**	16.6 (4.2) (79%)	16.1 (3.3) (77%)	−0.5	0.448
Treatments, 0–21 (*n* = 171)	13.4 (5.9) (64%)	14.1 (6.1) (67%)	12.6 (5.6) (60%)	−1.5	0.095	14.4 (5.0) (69%)	12.8 (6.3) (61%)	−1.6	0.092	14.2 (5.7) (69%)	12.3 (3.5) (60%)	−1.9	**0.041**
Self-management, 0–18 (*n* = 170)	12.3 (4.6) (69%)	12.9 (4.5) (72%)	11.8 (4.6) (65%)	−1.1	0.122	12.5 (4.0) (75%)	11.7 (4.8) (65%)	−1.9	**0.011**	12.3 (4.4) (69%)	12.4 (4.9) (68%)	+0.1	0.759
Support systems, 0–12 (*n* = 171)	5.6 (3.4) (47%)	6.0 (3.2) (50%)	5.2 (3.5) (43%)	−0.8	0.131	6.7 (3.1) (56%)	5.0 (3.3) (42%)	−1.8	**0.001**	5.7 (3.4) (49%)	5.6 (2.6) (46%)	−0.1	0.534
Total, 0–117 (*n* = 178)	66.5 (26.6) (57%)	70.9 (26.8) (61%)	62.0 (25.9) (53%)	−8.9	**0.026**	72.6 (24,7) (62%)	63.4 (27.1) (54%)	−9.2	**0.028**	68.9 (25.6) (60%)	64.1 (20.7) (55%)	−4.8	0.225

Bold text indicates *P*≤0.05.

ENAT: educational needs assessment tool.

When the total ENAT was explored, statistical differences were found. Women reported higher need compared with men (*P* = 0.02), and individuals with a disease duration of ≤2 years reported higher need compared with those with longer disease duration (*P* = 0.02).

### ENAT domains

Exploring each domain with the percentage of maximum domain score revealed different degrees of educational needs across the domains. The domains disease process (78%), self-management (69%) and treatments (64%) presented the highest need for education, whereas the feelings (56%), managing pain (48%), support systems (47%) and movement (41%) domains showed the lowest need for education. Women reported higher educational needs in the domains disease process, movement and feelings, compared with men (all *P* < 0.05; Table 3). Among participants with short disease duration (<2 years), more educational needs were reported for the domains disease process, self-management and support systems (all *P* < 0.05). Compared with those with higher education, patients who completed only primary school or high school had higher educational needs in the treatments and movement domains (all *P* < 0.05), but not in the total ENAT. When the total ENAT and domains were explored in relationship to age, diagnosis and disease activity, no significant differences were observed.

Those who acknowledged a current educational need (*n* = 67) scored significantly higher in all domains (all *P* ≤ 0.05) compared with those who denied a current educational need.

## Discussion

To the best of our knowledge, this is one of the first studies to use ENAT to explore the educational needs among patients with AAV. We found a distinctive need for education among all patients. The highest domain scores were found in the ENAT domains addressing the disease process, self-management and treatments. The results of this study highlights patients’ perception of needs and the importance of knowledge and education for patients with AAV. Women and those with recent disease onset seem to have slightly higher educational needs in some areas compared with men and those with longer disease duration.

In this study, the educational need captured by ENAT (57%) was comparable to previous studies in other rheumatic diagnoses, for RA and PsA (55%) [21], SpA (56%) [22] and SLE (56%) [23]. Our patients rated educational needs related to the domain concerning the disease process the highest, as previously shown by others [12]. In comparison to other rheumatic diseases, education regarding treatments was rated here as the third domain of most importance [12, 21, 22, 24]. Education on self-management was found to be important to patients with AAV, but not to the same extent as in other inflammatory diseases, such as SLE, SS and SpA [21, 22, 24]. This points to the fact that the need for education might vary between rheumatic diseases and might depend on the disease characteristics, symptoms and both short- and long-term need for therapy to control the disease. Thus, the demand for education regarding treatment here appeared to be more important compared with data reported in other diseases. This could depend on insufficient previous education provided from the health-care system, but also the fact that most AAV patients require both intense and long-lasting immunosuppressive therapy, which might necessitate both oral and written efforts to convey information on repeated occasions.

Furthermore, women reported a greater need for education than men. This is consistent with previous studies on the educational needs in patients with other rheumatic diseases [19, 21–23], with the exception of SS [24], as for patients with cancer [25, 26]. Studies have also shown that women are more inclined to seek information about their rheumatic disease in general compared with men [27]. Information on health information-seeking behaviour was not collected in our study and needs to be examined further in future studies.

We found a connection between educational needs and disease duration, in contrast to patients with SS [24] and SpA [21], where disease duration seems to have a low impact on educational needs. Another study on educational needs in AAV, using a breast cancer questionnaire adapted to fit AAV patients, did not find any association with disease duration [28]. Unlike our study, a relationship between educational needs and disease activity has been shown in other rheumatic diseases [22, 23], but has not been studied previously in AAV.

A strength of the present study is the number of participants, consisting of the majority of AAV patients in a rheumatological setting at a university hospital. The sample size is comparable to that of other studies using ENAT, despite the fact that AAV is a less common disease than RA or SpA [9, 19, 21]. This study covers both incident and prevalent cases, which makes it possible to examine educational needs at different stages of the disease. The overall response rate of the items in ENAT and the internal consistency of the total ENAT and domains were high, indicating that the questionnaire can be used in AAV. Given that the ENAT questionnaire was originally developed for RA, it might not be able to capture fully the educational needs of patients with AAV [21, 28]. However, all answers from the open text questions could be categorized into one of the existing ENAT domains. The answers revealed no new areas of interest, but ENAT could still contain questions that are less important to the individual patient. To conform or adapt the questionnaire to be of optimal use in AAV, more studies on educational needs in AAV patients should be conducted.

There are some limitations that need to be addressed. Information regarding how much or what type of education the participants had received before the inclusion time point was not available, nor were data regarding whether the patients had sought information about their illness themselves. Over the 10 years of the study data collection, the methods of information provision have changed and most probably increased and diversified. An updated data collection, preferably in conjunction with questions on how information was acquired, sought and preferred, could shed light on these issues. Furthermore, data on educational needs in patients with different disease manifestations (e.g. nephritis, lung manifestation) in AAV were not analysed, but might be of importance to tailor educational programmes in different disease phenotypes.

Using cross-sectional data only, no definitive assumptions can be made about whether the educational needs might change over time, although the results indicate that disease duration, rather than age or disease activity was important. Longitudinal data collections could also be used to explore how educational needs might change over time and in relationship to variation in disease activity. The ENAT could also be used to evaluate future targeted educational efforts and make education personalized in clinical practice. Our findings are in line with the BSR and BHPR guidelines [3], which state that AAV patients should receive ongoing personalized information about the disease and treatments and should be encouraged to engage in self-management.

## Conclusion

In conclusion, we found that 38% of our patients with AAV expressed a need for education, where the most important areas of educational needs were disease process, self-management and treatment. Women and patients with shorter disease duration should be given special consideration, because they were identified to have significantly higher educational needs in general. Further studies are needed to explore how educational needs might change over time and whether increased education can affect self-efficacy and adherence to treatment.

## Data Availability

The data underlying this article will be shared on reasonable request to the corresponding author.

## References

[1] Yates M , Watts RA, Bajema IM et al EULAR/ERA-EDTA recommendations for the management of ANCA-associated vasculitis. Ann Rheum Dis 2016;75:1583–94. 27338776 10.1136/annrheumdis-2016-209133

[2] Maheswaranathan M , Cantrell S, Eudy AM et al Investigating health literacy in systemic lupus erythematosus: a descriptive review. Curr Allergy Asthma Rep 2020;20:79. 33184709 10.1007/s11882-020-00978-6 PMC8261622

[3] Ntatsaki E , Carruthers D, Chakravarty K et al; BSR and BHPR Standards, Guidelines and Audit Working Group. BSR and BHPR guideline for the management of adults with ANCA-associated vasculitis. Rheumatology (Oxford) 2014;53:2306–9. 24729399 10.1093/rheumatology/ket445

[4] Herlyn K , Gross WL, Reinhold-Keller E. [Longitudinal effects of structured patient education programs for vasculitis patients]. Z Rheumatol 2008;67:206–10. 18431583 10.1007/s00393-008-0290-9

[5] Carpenter DM , Kadis JA, Devellis RF, Hogan SL, Jordan JM. The effect of medication-related support on the quality of life of patients with vasculitis in relapse and remission. J Rheumatol 2011;38:709–15. 21285174 10.3899/jrheum.100808 PMC3677822

[6] Thorpe CT , DeVellis RF, Blalock SJ et al Patient perceptions about illness self-management in ANCA-associated small vessel vasculitis. Rheumatology (Oxford) 2008;47:881–6. 18403403 10.1093/rheumatology/ken126 PMC4084613

[7] Mooney J , Poland F, Spalding N, Scott DG, Watts RA. ‘In one ear and out the other – it’s a lot to take in': a qualitative study exploring the informational needs of patients with ANCA-associated vasculitis. Musculoskeletal Care 2013;11:51–9. 22778039 10.1002/msc.1030

[8] Carpenter DM , DeVellis RF, Fisher EB et al The effect of conflicting medication information and physician support on medication adherence for chronically ill patients. Patient Educ Couns 2010;81:169–76. 20044230 10.1016/j.pec.2009.11.006 PMC2891323

[9] Hardware B , Lacey EA, Shewan J. Towards the developement of a tool to assess educational needs in patients with arthritis. Clin Eff Nurs 2004;8:111–7.

[10] Ndosi M , Bremander A, Hamnes B et al Validation of the educational needs assessment tool as a generic instrument for rheumatic diseases in seven European countries. Ann Rheum Dis 2014;73:2122–9. 23921996 10.1136/annrheumdis-2013-203461

[11] Ndosi M , Tennant A, Bergsten U et al Cross-cultural validation of the Educational Needs Assessment Tool in RA in 7 European countries. BMC Musculoskelet Disord 2011;12:110. 21609481 10.1186/1471-2474-12-110 PMC3126763

[12] Sierakowska M , Sierakowski S, Sierakowska J, Krajewska-Kułak E, Ndosi M. Pain, fatigue and functional disability are associated with higher educational needs in systemic sclerosis: a cross-sectional study. Rheumatol Int 2018;38:1471–8. 29497844 10.1007/s00296-018-3998-0 PMC6060785

[13] Ndosi M , Johnson D, Young T et al Effects of needs-based patient education on self-efficacy and health outcomes in people with rheumatoid arthritis: a multicentre, single blind, randomised controlled trial. Ann Rheum Dis 2016;75:1126–32. 26162769 10.1136/annrheumdis-2014-207171 PMC4893097

[14] Watts R , Lane S, Hanslik T et al Development and validation of a consensus methodology for the classification of the ANCA-associated vasculitides and polyarteritis nodosa for epidemiological studies. Ann Rheum Dis 2007;66:222–7. 16901958 10.1136/ard.2006.054593 PMC1798520

[15] Antovic A , Svensson E, Lövström B et al Venous thromboembolism in anti-neutrophil cytoplasmic antibody-associated vasculitis: an underlying prothrombotic condition? Rheumatol Adv Pract 2020;4:rkaa056. 33215056 10.1093/rap/rkaa056 PMC7661844

[16] Elo S , Kyngäs H. The qualitative content analysis process. J Adv Nurs 2008;62:107–15. 18352969 10.1111/j.1365-2648.2007.04569.x

[17] Luqmani RA , Bacon PA, Moots RJ et al Birmingham Vasculitis Activity Score (BVAS) in systemic necrotizing vasculitis. QJM 1994;87:671–8. 7820541

[18] Theme report 2017:4. Statistics Sweden. 2017. https://www.scb.se/contentassets/8470acff99c54f21bd4aba8ca058cb5b/uf0549_2015a01_br_a40br1704.pdf.

[19] Meesters JJ , Vliet Vlieland TP, Hill J, Ndosi ME. Measuring educational needs among patients with rheumatoid arthritis using the Dutch version of the Educational Needs Assessment Tool (DENAT). Clin Rheumatol 2009;28:1073–7. 19449083 10.1007/s10067-009-1190-3 PMC2721136

[20] Terwee CB , Bot SD, de Boer MR et al Quality criteria were proposed for measurement properties of health status questionnaires. J Clin Epidemiol 2007;60:34–42. 17161752 10.1016/j.jclinepi.2006.03.012

[21] Drăgoi RG , Ndosi M, Sadlonova M et al Patient education, disease activity and physical function: can we be more targeted? A cross sectional study among people with rheumatoid arthritis, psoriatic arthritis and hand osteoarthritis. Arthritis Res Ther 2013;15:R156. 24286444 10.1186/ar4339 PMC3978882

[22] Haglund E , Bremander A, Bergman S, Larsson I. Educational needs in patients with spondyloarthritis in Sweden – a mixed-methods study. BMC Musculoskelet Disord 2017;18:335. 28768510 10.1186/s12891-017-1689-8 PMC5541660

[23] Zirkzee EJ , Ndosi ME, Vliet Vlieland TP, Meesters JJ. Measuring educational needs among patients with systemic lupus erythematosus (SLE) using the Dutch version of the Educational Needs Assessment Tool (D-ENAT). Lupus 2014;23:1370–6. 25059487 10.1177/0961203314544188

[24] Schouffoer A , Ndosi ME, Vliet Vlieland TP, Meesters JJ. The educational needs of people with systemic sclerosis: a cross-sectional study using the Dutch version of the Educational Needs Assessment Tool (D-ENAT). Rheumatol Int 2016;36:289–94. 26321625 10.1007/s00296-015-3352-8 PMC4723617

[25] Mistry A , Wilson S, Priestman T, Damery S, Haque M. How do the information needs of cancer patients differ at different stages of the cancer journey? A cross-sectional survey. JRSM Short Rep 2010;1:1. 21103122 10.1258/shorts.2010.010032 PMC2984359

[26] Matsuyama RK , Kuhn LA, Molisani A, Wilson-Genderson MC. Cancer patients' information needs the first nine months after diagnosis. Patient Educ Couns 2013;90:96–102. 23058682 10.1016/j.pec.2012.09.009

[27] Cooksey R , Brophy S, Husain MJ et al The information needs of people living with ankylosing spondylitis: a questionnaire survey. BMC Musculoskelet Disord 2012;13:243. 23227937 10.1186/1471-2474-13-243 PMC3553011

[28] Mooney J , Spalding N, Poland F et al The informational needs of patients with ANCA-associated vasculitis—development of an informational needs questionnaire. Rheumatology (Oxford) 2014;53:1414–21. 24625507 10.1093/rheumatology/keu026 PMC4103516

